# CD21^int^ CD23^+^ follicular B cells express antigen‐specific secretory IgM mRNA as primary and memory responses

**DOI:** 10.1111/imm.12724

**Published:** 2017-03-16

**Authors:** Qing‐Hai Meng, Harry N. White

**Affiliations:** ^1^ Molecular Immunology UnitInstitute of Child HealthUniversity College LondonLondonUK; ^2^ Department of BiosciencesUniversity of ExeterExeterUK

**Keywords:** B cells, follicular, memory, repertoire

## Abstract

CD21^int^ CD23^+^ IgM^+^ mouse follicular B cells comprise the bulk of the mature B‐cell compartment, but it is not known whether these cells contribute to the humoral antibody response. We show using a direct RT‐PCR method for antigen‐specific VH, that FACS‐sorted mouse CD21^int^ CD23^+^ B cells express specific secretory IgM VH transcripts in response to immunization and also exhibit a memory response. The secretory IgM expressed is distinct from the IgG expressed by cells of this phenotype, which we also analyse here, having a distinct broader distribution of CDR‐H3 sequences and zero or low levels of somatic mutation in the region analysed. These results imply that cells of the CD21^int^ CD23^+^ phenotype have distinct IgM^+^ and IgG^+^ populations that contribute directly to the humoral antibody and memory responses by expressing antigen‐specific secretory immunoglobulin. We also argue that the more diverse CDR‐H3 sequences expressed by antigen‐experienced IgM^+^ CD21^int^ CD23^+^ follicular B cells would place them at the bottom of a recently hypothesized memory B‐cell hierarchy.

**Abbreviations**
C6: the 6 amino‐acid CDR‐H3 of particular VHOx‐1 transcripts
CSA: chicken serum albumin
phOx: phenyl‐oxazolone

## Introduction

Recent studies on antibody responses suggest that the memory B‐cell compartment is diverse and may contain cells not previously considered to be memory B cells, as they have some characteristics of naive cells.^1^

The bulk of the mouse mature ‘naive’ B‐cell compartment is comprised of CD21^int^ CD23^+^ follicular B cells that express IgM and IgD. These cells are able to re‐circulate and home to B‐cell follicles in secondary lymphoid tissue where they are well placed to initiate antibody responses.^2^, ^3^, ^4^ Follicular B cells show functional plasticity; as well as their role in initiating T‐cell‐dependent responses and affinity maturation in germinal centres, they also participate in T‐cell‐independent responses in the bone marrow,^5^ and may act as marginal zone B‐cell precursors.^6^

B‐cell receptor signalling is important in the development and survival of mature B cells,^7^, ^8^, ^9^, ^10^, ^11^ and the strength of signalling is proposed as a factor during follicular B‐cell development.^4^ Self antigens,^12^ and perhaps gut flora,^12^, ^13^ have been proposed as drivers of marginal zone B‐cell development, and it has recently been shown that foreign antigen can select certain VH into the marginal zone antibody repertoire,^14^ but the situation with follicular B‐cell selection is less clear.

The CD21^int^ CD23^+^ IgM^+^ population has long been assumed to be naive. Although follicular cells usually have a lifespan of several weeks this is probably extendable depending on homeostatic forces and quality of B‐cell receptor stimulation.^3^, ^15^, ^16^, ^17^ It is not clear, therefore, how smoothly distributed the clonality is in the follicular population; whether there are as many different clones as cells – perfectly smooth – or whether some clones are larger or have longer lifespans, perhaps due to foreign antigen stimulation.

Recent studies on memory B cells have shown that they can be of either IgM or IgG isotype. Memory cells may also reside in the follicular areas as well as the marginal zone of the spleen^18^ and may not have gone through germinal centres.^19^, ^21^ Because of this diversity in the B‐cell memory compartment,^18^, ^19^, ^20^, ^21^ and the likelihood that this compartment is organized as a hierarchy to maximize adaptability,^19^, ^21^, ^22^, ^23^ and includes IgM^+^ IgD^+^ cells with few or no VH mutations,^19^ a question has to be asked about what relationship CD21^int^ CD23^+^ IgM^+^ follicular B cells have to the memory compartment, and whether perhaps some of them represent the bottom, most ‘naive‐like’^22^ part of the memory hierarchy. A current definition of a memory cell involves it having experienced antigen and subsequently being in a resting state, and then being capable of secondary activation.^18^, ^20^ Follicular B cells could fulfil these criteria if foreign antigens stimulated increased recruitment, division or survival without a subsequent differentiation further into the memory/effector response. This would form a memory pool of the sort described for T‐independent antigens, that is not necessarily long lived and is caused by an increase in precursor frequency rather than affinity.^20^

Antigen‐specific follicular B cells are rare, being present at a frequency in the order of 10^−4^ in naive mice.^19^ For certain antigens it is possible to use RT‐PCR to analyse antibody expression, and this represents an ideal approach to directly assess specific antibody expression by rare cells in a large population. We have previously reported the use of immunoglobulin RT‐PCR to detect expression of phenyl‐oxazolone (phOx) ‐specific VH.^14^, ^24^ This method is an adaptation of the method of ‘spectratyping’.^25^ A further feature of the phOx response is that all reported hybridomas and all secretory IgG we detect by RT‐PCR, with the VHOx‐1 heavy chain, contain a particular CDR‐H3 motif containing a DXG sequence, the basis of the Ox idiotype.^24^, ^26^, ^27^ This DXG motif is, therefore, a useful marker for the presence of presumptive higher affinity VH. The phOx‐specific DXG‐motif‐containing CDR‐H3 is always six amino acids long. In the RT‐PCR analysis reported in this study we refer to VHOx‐1 transcripts encoding CDR‐H3s of this length as C6 transcripts. As the modal VHOx‐1 CDR‐H3 length is ten, C6 transcripts appear four codon units down from these on gels. The phOx/RT‐PCR method is highly reproducible, detecting up‐regulated IgM and/or IgG transcripts in every immunized mouse of the appropriate IgH haplotype.^14^, ^24^, ^28^ Further, for reasons dealt with in depth elsewhere,^14^ the RT‐PCR method used is independent of known mechanisms of PCR bias.

To address the question of whether the CD21^int^ CD23^+^ IgM^+^ splenic B‐cell population shows any memory capacity, we have conducted an analysis of secretory VHOx‐1 IgM expression on FACS‐sorted cells at various times after phOx immunization/re‐challenge. We show that CD21^int^ CD23^+^ B cells express a diverse repertoire of secretory IgM mRNA in unimmunized mice, that antigen priming induces a significant up‐regulation of specific secretory IgM expression by these cells, and that they also show a memory/recall response. By analysing IgG expression in the same population we also show that this IgM memory response is distinct from that expressed by CD21^int^ CD23^+^ IgG^+^ cells.

## Materials and methods

#### Mice

All mice were female BALB/c (Harlan, Bicester, UK), 6–8 weeks old at day 0. Age‐matched controls were 14 weeks old. Mice were maintained in the Institute of Child Health Biological Services Unit in compliance with UK Home Office Animals (Scientific Procedures) Act 1986 Guidelines and experiments were performed under the protocols of a UK Home Office Animal Project license # 70/05954, approved by the animal experimentation ethics committee of the UCL/Institute of Child Health.

#### Immunization

The hapten‐carrier phOx‐CSA was made as described previously.^28^, ^29^ For primary immunizations (day 0) 100 μg of alum‐precipitated phOx‐CSA with 10^9^ heat‐killed pertussis was injected intraperitoneally. For boosting (day 50) 100 μg soluble phOx‐CSA in PBS was injected iintraperitoneally.

#### FACS sorting

Eighty‐six per cent of a whole sieved spleen cell suspension was incubated in 1 ml medium with 10% fetal calf serum and 1/50 Fc Block (BD Pharmingen, Oxford, UK) for 15 min on ice, before incubation with 1/50 dilutions of appropriate antibodies for 30 min on ice. Most antibodies were obtained from BD Pharmingen including FITC‐conjugated anti‐CD21 (clone 7G6) and phycoerythrin‐conjugated anti‐CD23 (clone B3B4). Cy‐chrome (phycoerythrin‐Cy5) ‐conjugated anti‐CD19 (clone 6D5) was obtained from AbD Serotec/BioRad (Kidlington, Oxford, UK). Cells were washed and sorted on a Beckman‐Coulter Altra. Between 1 × 10^6^ and 3 × 10^6^ follicular B cells were recovered from each sample.

#### RNA extraction/RT‐PCR

This method has been described elsewhere.^24^ Briefly, total RNA extraction was performed using the RNA Bee reagent (Amsbio, Abingdon, UK) according to the manufacturer's instructions. Twenty per cent of the RNA obtained from a whole cell‐sort sample was denatured at 96° for 3 min with 0·5 μg oligo‐dT and placed on ice before reverse transcription in a 50‐μl reaction for 1 hr at 42° with 1× RT buffer, 0·2 mm dNTPs, 1 mm dithiothreitol, 800 Uml‐1 RNAsin (Promega, Southampton, UK) and 200 U Superscript II reverse transcriptase. After heat inactivation, reactions were digested for 20 min with 2 U RNase H (all Thermo Fisher, Paisley, UK except where indicated).

First‐round PCR was done with 2 μl of cDNA reaction with standard reaction conditions using the manufacturer‐supplied buffer with Hotstart Pfu turbo polymerase (Agilent Stratagene, Stockport, UK) and 25 pmol of the appropriate primer in a 50‐μl reaction for 34 cycles with the following cycling: 96°, 30 seconds; 59°, 30 seconds; 72°, 2 min; preceded by an incubation at 96° for 3 min. All primers used are described elsewhere.^24^

#### Run‐offs

For each single run‐off reaction equivalent 2 pmol nested isotype‐specific primer was end‐labelled with 1 μCi *γ* ^33^P dATP (2500 Ci/mmol, GE Healthcare, Chalfont St Giles, UK) using the T4 polynucleotide kinase labelling system (GE Healthcare). The reaction was sodium acetate/ethanol precipitated and washed with 75% ethanol before use. Run‐off reactions were performed for 12 cycles in a 20‐μl volume with Hotstart Pfu‐turbo polymerase and buffer as before using 2 μl of the first‐round PCR product with the following cycling: 96°, 30 seconds; 60°, 30 seconds; 72°, 1 min. After cycling reactions, samples were treated as for standard sequencing gel electrophoresis and run on 5% acrylamide/urea gels, dried and exposed to X‐ray film (Kodak XAR).

Run‐offs for quantification on the capillary DNA analyser were prepared as follows: 2 μl of first‐round PCR was put into a total run‐off reaction volume of 10 μl, using buffers as above, with 1 pmol of FAM‐labelled nested run‐off primer (Eurofins/MWG, Germany) and cycled 10 times with the parameters as above. One microlitre of run‐off was diluted into 12 μl H_2_O and 0·35 μl of ROX size standards (GE Healthcare) were added. Samples were analysed on an GE Megabace 500 using genetic profiler software (GE Healthcare). All primers used are described elsewhere.^24^

#### CDR‐H3 sequence determination

For PAGE purification of ‘C6’ transcripts quadruple‐sized run‐off samples were loaded on triple‐width wells, electrophoresed and located by autoradiography. Bands were cut out from the dried gel with fine scissors, soaked for 10 min in 100 μl of water, heated to 100° for 15 min and centrifuged at 21,000 ***g*** for 2 min. DNA was recovered from the supernatant by addition of 10 μg of linear polyacrylamide and sodium acetate/ethanol precipitation on dry ice. After centrifugation, the pellet was washed with 75% ethanol, air‐dried and re‐dissolved in 10 μl of water. Six microlitres of this was used in a 50‐μl, 15‐cycle PCR with Hot‐start Pfu turbo polymerase and the manufacturer‐supplied buffer with conditions as above and the OXC2 and appropriate nested isotype‐specific primer that was also used in the run‐off, with the following cycling: 94°, 30 seconds; 59°, 30 seconds; 72°, 1 min. Two microlitres of this product was Zero‐Blunt TOPO‐TA cloned, transformed into *Escherichia coli* TOP‐10 and plated out on selective media according to the manufacturer's instructions (Thermo Fisher, Paisley, UK). Individual colonies were picked and directly amplified for 25 cycles with Hot‐start Pfu‐turbo (Agilent Stratagene, UK) conditions as above, using the OXC2 and appropriate nested isotype‐specific primer. One microlitre of this product was sequenced directly using the ABI Big‐Dye kit (Life Technologies) and sequenced on a GE Megabace 500 DNA analyser. Twenty‐four colonies were picked and sequenced for each sample. Only sequences with clean reads and six‐amino‐acid CDR‐H3s, that translated correctly from the beginning of VHOx‐1 FR3 to GQG in JH were used. For VHOx‐1 IgG CDR‐H3 sequence determination we adopted a direct approach. As VHOx‐1 IgG is not detected by RT‐PCR in unimmunized mice, and as the bulk of it in immunized mice is C6 transcripts, gel purification after first‐round PCR is not necessary. The cDNA samples were amplified as in ‘RT‐PCR’ above using the VHOx‐1primer and the common IgG secretory form redundant primers G13sec: tcatttaccaggrgagygrga/G2Absec: tcatttacccggagwccggga which were used at equimolar concentrations, and common IgG membrane form primer GMEM: caggaagaggctgatgaagatgg. These samples were then subject to a nested amplification, as in this section above, with a primer common for the CH1 of all IgG subclasses acacyrctggacagggmtcca, then TOPO cloned and sequenced as above.

## Results

**Figure 1:**
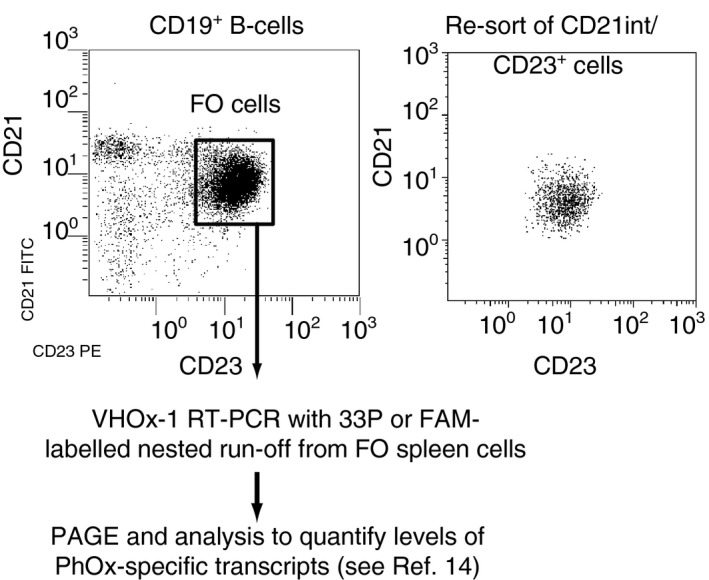
FACS gating and workflow for RT‐PCR analysis of specific antibody expression. Cell suspensions were sorted for CD19^+^ CD21^int^ CD23^+^ cells when appropriate. Upper left hand panel shows a typical sort and the follicular cell gate used. Upper right hand panel shows a re‐sort of CD21^int^ CD23^+^ cells after sample sort was completed.

Figure 1 shows the FACs gating and work flow to analyse levels of antigen‐specific transcripts in follicular B cells or whole spleen cells. This method is dealt with in more detail elsewhere.^14^ Whole spleen cells or FACS‐sorted cells were subject to secretory or membrane form‐specific, isotype‐specific, VH‐specific RT‐PCR. Amplified transcripts were then subject to a nested isotype‐specific FAM‐ or ^33^P‐labelled run‐off, to label the shorter FR3‐CDR‐H3‐JH‐CH1 fragment for quantification on a DNA analyser or visualization and DNA cloning.

### The splenic secretory IgM response to phox

**Figure 2:**
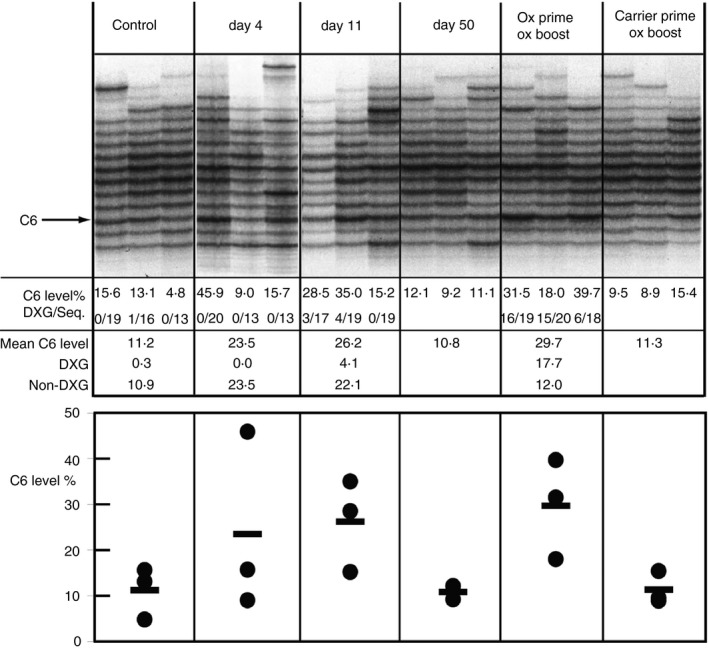
The VHOx‐1 secretory IgM response to phenyl‐oxazolone (phOx) at the whole spleen level. VHOx‐1 secretory IgM RT‐PCR of whole spleen cell suspension cDNA. Cells from single mouse spleen per sample track. Three samples per group from one representative experiment. Control, unimmunized; day 4: Four days after immunization with phOx‐CSA/adjuvant; day 11: 11 days after immunization with phOx‐CSA/adjuvant; day 50: 50 days after immunization with phOx‐CSA/adjuvant; ox prime/ox boost: re‐immunized with soluble phOx‐CSA 50 days after priming with phOx‐CSA/adjuvant, samples collected 4 days later; carrier‐prime/ox boost: re‐immunized with soluble phOx‐CSA 50 days after priming with CSA/adjuvant, samples collected 4 days later. The C6 band, containing phOx‐specific transcripts with six amino‐acid CDR‐H3s, is indicated with an arrow. Numerical data derived as described ref. 14 C6 level %, the level of C6 transcripts in that sample as a percentage of the levels of all other VHOx‐1 transcript lengths. DXG/seq, the number of sequences in that C6 band containing the DXG motif out of all sequences from that band. Mean C6 level, the mean level of C6 transcripts in that group. DXG, the mean level of DXG motif containing C6 transcripts in that group as a percentage of all other VHOx‐1. To calculate this mean value, the individual sample DXG values were calculated first, using individual C6% × DXG/Seq values, and then averaged; Non DXG, the mean level of C6 transcripts without the DXG motif expressed as a percentage of all other VHOx‐1. Lower panel shows individual C6 levels plotted (dots) and the mean for each group (solid bar), and panels correspond to those above.

Groups of three mice were immunized with phOx‐CSA in an alum/pertussis adjuvant and analysed 4, 11 and 50 days later. A further group was re‐immunized with soluble phox‐CSA at day 50 and analysed 4 days later. Three unimmunized age‐matched controls and three memory controls primed with CSA/alum/pertussis and re‐immunized with soluble phOx‐CSA were also included. The upper panel of Fig. 2 shows the transcript profiles, the values for C6 transcript levels in each sample and the frequency of the DXG motif within each C6 transcript ‘band’.

Expressing C6 transcript levels as the percentage of transcript levels in all ‘bands’ in the VHOx‐1 repertoire, C6/(totalVHOx1‐C6), provides a measure independent of known mechanisms of PCR bias and allows a direct quantification of C6 transcript levels. When the C6 gel band is cloned from ^33^P‐labelled gels and the CDR‐H3 sequences in it determined, a measure is also possible of the separate levels of phOx‐induced C6 transcript with and without the DXG CDR‐H3 sequence motif.

Antigen priming induced a change in C6 transcript levels, increasing from 11·2% to 26·2% by day 11. Sequence analysis of the transcripts showed that about three‐quarters of this response was comprised of transcripts without the DXG motif, compared with secretory IgG transcripts from the spleen at this time‐point, which all contain the motif.^24^ By day 50, C6 secretory IgM transcripts had returned to pre‐immune levels. Re‐challenge with phOx induced a strong secondary, memory‐type response compared with the C6 levels seen in the carrier‐primed/phOx boosted group. This effect formed the basis of our previous report of IgM memory.^24^ Here, we also observed that C6 transcripts in the secondary response contained an increase in the frequency of DXG motifs compared with the antigen‐primed samples (Fig. 2 and Table 1). Also, comparing the levels of non‐DXG and DXG in C6 transcripts between boost and pre‐immune groups showed that the uplift in expression after boosting can be mostly accounted for by DXG motif transcripts.

**Table 1:** Frequency of, and mutation in, the DXG motif transcripts up‐regulated in response to phenyl‐oxazolone

	Prime day 11		Boost	
	% DXG	% mutation in DXG	% DXG	% mutation in DXG
Whole spleen				
Secretory IgM	25·3	0·0 (0/7)	94·1	0·35 (15/37)
CD21^int^ CD23^+^ cells				
Secretory IgM	4·2	0·0 (0/2)	63·6	0·28 (7/21)
Membrane IgM	64·2	0·32 (11/29)	59·3	0·39 (6/13)
Secretory IgG	100·0 (34/34)	0·2 (9/34)	100·0 (30/30)	1·25 (44/30)
Membrane IgG	95·2 (20/21)	0·47 (11/20)	100·0 (40/40)	1·38 (65/40)

**Figure 3:**
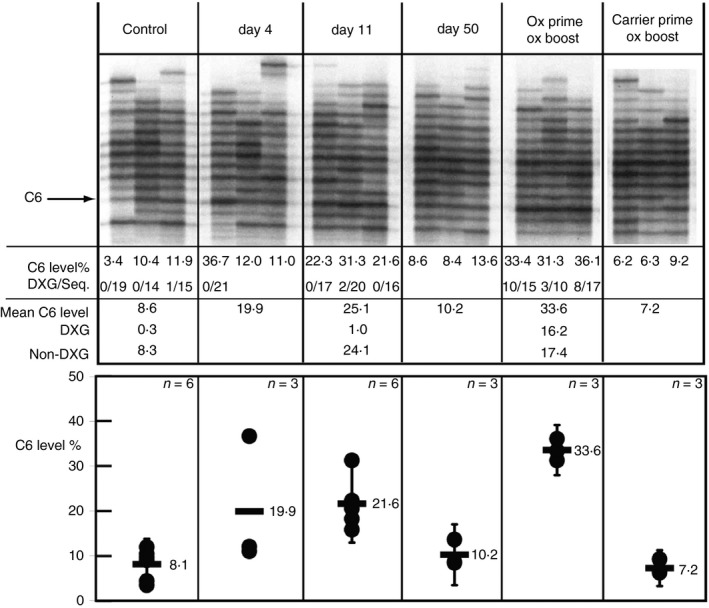
The VHOx‐1 secretory IgM response to phenyl‐oxazolone (phOx) in the CD21^int^ CD23^+^ population. VHOx‐1 secretory IgM RT‐PCR of sorted CD21^int^ CD23^+^ cell cDNA. Cells were from single mouse per sample track, three samples per group. For statistical calculations a further three samples from an independent experiment for control and day 11 post‐immunization samples were obtained for data in lower panel. The extra C6%‐level data‐points not shown in upper panel, from the second experiment were: Control: 9·0, 4·3, 9·6; day 11: 15·8, 20·5, 18·2. Sample time‐points, derivation of numerical data and annotation as described in Fig. 2. Lower panel: shows individual C6 levels plotted (dots), the mean for each group (solid bar) which may differ from upper panel because of the inclusion of extra samples, and the 95% confidence intervals for the mean, calculated according to ref. 33; for *n* = 3 or 6 the 95% CI = 4 × SEM. Confidence intervals not shown for day 4 samples because only one individual showed response.

Estimated frequency in % of the DXG motif in transcripts expressed above pre‐immune levels; and level of somatic mutation in the FR3 region of the VHOx‐1/DXG sequences. Day 11: 11 days after immunization with phOx‐CSA/adjuvant, boost: re‐immunized with soluble phOx‐CSA 50 days after priming with CSA/adjuvant, samples collected 4 days later. % DXG: Proportion of phenyl‐oxazolone (phOx) ‐induced transcripts that contain DXG motif. For estimating % DXG in phOx‐induced transcripts, IgM values were calculated from data in Figs 2 and 3, except membrane IgM data which is derived from ref. 13 by subtracting the mean pre‐immune values of DXG and non‐DXG transcript levels from the respective mean values for each experimental group and then calculating what percentage were DXG/non‐DXG. For each experimental group, between 42 and 57 DNA sequences were analysed in total for DXG from the three independent samples shown in these figures. VHOx‐1/C6 IgG is not readily detectable in pre‐immune samples so all is assumed phOx‐induced. Figures in brackets by IgG values show DXG/number of total sequences analysed, a pool from the three independent samples in each group.

% mutation in DXG: Figures show % somatic mutation in the FR3 region of VHOx‐1/C6 for all transcripts in group with DXG motif. Figures in brackets show number of mutations/number of sequenced 117 bp FR3 regions.

In summary, when analysed at the whole spleen level, the primary secretory IgM response to phOx involved expression of C6 transcripts mostly lacking the DXG motif, whereas in the secondary response DXG‐containing transcripts largely accounted for the up‐regulated C6 levels. Although, as detected here, there was not a difference between overall C6 transcript levels at day 11 after priming and after boosting, 26·2% versus 29·7%, the secondary response was qualitatively different in containing higher levels of the presumptive higher‐affinity DXG motif, and levels of C6 were much higher after boosting than observed in the carrier‐primed/phOx boosted memory control.

### Cells with the CD21^int^ CD23^+^ phenotype express secretory IgM mRNA

FAC‐sorted CD21^int^ CD23^+^ cells were obtained from the same spleen samples analysed above and subject to VHOx‐1 secretory IgM RT‐PCR. The results and analysis are shown in Fig. 3. The CD21^int^ CD23^+^ population expressed a diverse repertoire of secretory IgM transcripts in unimmunized mice. Considering the abundance of this population, comprising about 80% of splenic CD19^+^ B cells, and the diversity of the repertoire, this observation suggested that many CD21^int^ CD23^+^ B cells contribute to the plasma IgM pool.

### CD21^int^ CD23^+^ B cells express particular secretory IgM transcripts in response to immunization

Figure 3 shows that phOx stimulated a significant increase in C6 secretory IgM transcript expression in the CD21^int^ CD23^+^ population after 11 days (*P* = 0·0004, Students *t*‐test, see Fig. 3 legend). The increase in C6 transcripts is accounted for by expression of C6 without the DXG motif. Table 1 shows a summary of DXG frequency in IgM and IgG C6 transcripts, up‐regulated in response to phOx immunization, in CD21^int^ CD23^+^ cells. Although few (4·2%) antigen‐priming‐induced secretory IgM transcripts contained the motif, all tested secretory IgG transcripts from the same sorted population were DXG^+^. We have also included membrane IgG CDR‐H3 data for this population, which confirmed that memory‐type membrane IgG^+^, DXG‐expressing cells were within the CD21^int^ CD23^+^ phenotype 11 days after phOx priming.

Hence, at 11 days after antigen priming, not all antigen‐responding CD21^int^ CD23^+^ IgM^+^ cells had differentiated and/or lost this phenotype. Those cells that retained the phenotype expressed secretory IgM with the same VH but distinct CDR‐H3s to those seen in the IgG response, which was also running at this time‐point.

It is clear comparing the data and profiles from Figs 2 and 3, that the bulk of the VHOx‐1/C6 secretory IgM response to phOx detected by this method was produced by CD21^int^ CD23^+^ cells. This population comprises around 80% of CD19^+^ splenic B cells, FACs sorting them did not obviously change the profiles compared with whole spleen. We discuss the implications of this below. Figure 3 also shows that by 50 days after phOx priming, levels of C6 secretory IgM transcripts had fallen to pre‐immune levels in these cells.

### The CD21^int^ CD23^+^ IgM^+^ B‐cell population expresses secretory IgM transcripts as a memory response

Figure 3 shows that rechallenge with phOx stimulated a large increase in VHOx‐1/C6 secretory IgM, 4 days after re‐challenge. Further, this response was significantly larger (based on 95% confidence intervals) than that seen in the carrier‐primed/phOx boosted samples, 33·6% compared with 7·2%.

Analysis of the transcripts in the boosted samples from CD21^int^ CD23^+^ cells showed that there was a mixture of DXG and non‐DXG. Table 1 shows that up‐regulated transcripts were approximately two‐thirds DXG and one‐third non‐DXG. Table 1 also shows that as with phOx‐primed cells, all the secretory IgG transcripts from phOx‐boosted CD21^int^ CD23^+^ cells had DXG motif CDR‐H3s, showing again that the secretory IgM response by this cell population involved a different subset of VHOx‐1 sequences. This observation was further supported by comparing the levels of somatic mutation in the framework‐3 region (FR3) from DXG^+^ C6 transcripts between secretory IgM and secretory IgG in boosted mice, see Table 1. After boosting, CD21^int^ CD23^+^ B cells expressed secretory IgG with a four‐fold higher level of somatic mutation in FR3 compared with that seen in secretory IgM expressed by this population, 1·25% versus 0·28%. This distribution of somatic mutation, higher in IgG‐expressing cells, confirms that the CD21^int^ CD23^+^ cells expressing IgM and IgG were distinct populations, at the level of VH sequences.

These observations also showed that not all primed, antigen‐responsive CD21^int^ CD23^+^ IgG^+^ cells lose this phenotype after antigen re‐challenge. We have also included membrane IgG CDR‐H3 data for this population, which showed that memory‐type, membrane IgG‐expressing, DXG‐expressing cells remain within the CD21^int^ CD23^+^ phenotype 4 days after boosting (Table 1).

## Discussion

Using a simple immunization strategy and direct RT‐PCR for antigen‐specific VH, we have shown that CD21^int^ CD23^+^ B cells express antigen‐specific secretory IgM mRNA and exhibit a memory response. The IgM expressed in these circumstances is distinct from the IgG expressed by cells of the same phenotype both by composition of the CDR‐H3 and by level of somatic mutation in the FR3 region, indicating the presence of two different antigen‐experienced sub‐populations within the larger CD21^int^ CD23^+^ phenotype. Further, the secretory IgM memory response of these cells is qualitatively different from the primary response by this population, containing a higher frequency of the presumptive high‐affinity DXG motifs and is also expressed at higher level compared with the carrier‐primed memory control group. We note that the secretory IgM VHOx‐1/C6 primary response reported here is higher than in one of our previous studies,^24^ and conclude that this is due to the higher antigen dose of 100 μg used in the current study compared with 30 μg previously. We used this higher dose of antigen as we theorized that it was more likely to stimulate responses in the follicular cell population, perhaps by cells with lower affinity VH.

For particular cells in the follicular cell pool to be stimulated by foreign antigen and express specific secretory IgM, but not undergo a net decrease in cell numbers, we suggest that they are probably expressing B‐cell receptor that gives an appropriate level of stimulation but does not bind antigen strongly enough to drive them into a T‐dependent response, but does stimulate the B‐cell receptor enough to increase recruitment into the follicular pool or increase cell division or survival. In the diverse repertoire of CD21^int^ CD23^+^ IgM one would expect some VH with these characteristics of intermediate affinity to a challenging antigen, and the prediction would be that these would be distinct sequences from those found in IgG. This is what we have observed in this study, as the frequency of the DXG motif differs between the IgM^+^ and IgG^+^ populations.

As well as the difference in the presence of the DXG motif between IgM and IgG transcripts, the differing levels of somatic mutation that we have observed in the FR3 region of VHOx‐1 after boosting, 0·28% in secretory IgM and 1·25% in secretory IgG expressing cells, confirms that the CD21^int^ CD23^+^ cells responding to re‐challenge by expressing IgM and IgG are distinct populations. It is not possible to know, however, whether this class switching happened before or after boosting, as antigen re‐challenge could have stimulated rapid class switching from IgM by cells expressing the higher affinity and/or most mutated VH.

By 11 days after priming the T‐independent IgM response has peaked and is fading.^30^, ^31^ We suggest, both because of the use of the VHOx‐1/C6 VH and the dynamics of the response, that the effects we are observing are the T‐independent beginnings of the T‐dependent response to phOx. Current models of the memory compartment suggest a structured hierarchy with longer‐lived less‐mutated IgM memory cells at the root.^19^, ^21^, ^22^ This architecture allows maximum adaptability, whereby in response to pathogenic epitope–escape variants, IgM^+^ cells can enter germinal centres and undergo affinity maturation towards a variant epitope faster than naive cells. It is not unreasonable to propose that a temporarily expanded clone of CD21^int^ CD23^+^ IgM^+^ B cells could provide a similar advantage when pathogen epitopes were too greatly mutated to bind to immunoglobulin on established IgM^+^ memory cells. This would make follicular cells the most ‘naive‐like’^22^ part of the B‐cell memory compartment.

This study has also revealed another interesting aspect of the expression of the VHOx‐1//C6‐containing antibody in response to phOx. Both in our previous studies^24^ and as reported here, there is little evidence for a VHOx‐1/C6 IgM plasma cell response. The similarity between Figs 2 and 3 is notable, and implies that all or most of the VHOx‐1/C6 secretory IgM that we detect is follicular cell derived. The uplift in splenic secretory IgM C6 after priming and boosting, although higher than controls and carrier primed controls is moderate when compared with IgG,^24^ and undiminshed when only follicular cells are analysed, Fig. 3. In unimmunized animals VHOx‐1 IgG1 is hard to detect, but after boosting IgG1 C6 transcripts alone saturate the RT‐PCR.^24^ These observations imply that all or almost all of the non‐follicular IgM response to phOx, including any T‐independent part, is not using the VHOx‐1/C6 combination at the observed time‐points. This is consistent with earlier studies^26^ reporting that VHOx‐1 IgM hybridomas are rare and that depending on the time‐point and number of immunizations, 25–75% of the phOx response does not use VHOx‐1. Only one mouse showed a VHOx‐1/C6 response at day 4 after immunization, which still partitioned to the CD21^int^ CD23^+^ population and so was expressed by cells with the follicular phenotype. At this time, marginal zone B‐cell‐derived^31^ and early follicular B‐cell‐derived^32^ antibody forming cell (AFC) formation has peaked, this also suggests that these parts of the IgM antibody response to phOx are not commonly using the VHOx‐1/C6 combination.

This would explain why, when comparing the data and profiles from Figs 2 and 3, that the bulk of the VHOx‐1/C6 secretory IgM response to phOx detected by this method is produced by CD21^int^ CD23^+^ cells. Finally, we conclude this because the follicular cell population comprises around 80% of CD19^+^ splenic B cells, and enriching for them does not obviously change the VHOx‐1/C6 expression profiles compared with whole spleen.

## Disclosures

The authors declare no competing financial interests.
